# Identification of Highly Methylated Genes across Various Types of B-Cell Non-Hodgkin Lymphoma

**DOI:** 10.1371/journal.pone.0079602

**Published:** 2013-11-19

**Authors:** Nicole Bethge, Hilde Honne, Vera Hilden, Gunhild Trøen, Mette Eknæs, Knut Liestøl, Harald Holte, Jan Delabie, Erlend B. Smeland, Guro E. Lind

**Affiliations:** 1 Department of Immunology, Institute for Cancer Research, Oslo University Hospital, Oslo, Norway; 2 Centre for Cancer Biomedicine, University of Oslo, Oslo, Norway; 3 Department of Cancer Prevention, Institute for Cancer Research, Oslo University Hospital, Oslo, Norway; 4 Department of Pathology, Oslo University Hospital, Oslo, Norway; 5 Department of Informatics, University of Oslo, Oslo, Norway; 6 Department of Oncology, The Norwegian Radium Hospital, Oslo University Hospital, Oslo, Norway; Bellvitge Biomedical Research Institute (IDIBELL), Spain

## Abstract

Epigenetic alterations of gene expression are important in the development of cancer. In this study, we identified genes which are epigenetically altered in major lymphoma types. We used DNA microarray technology to assess changes in gene expression after treatment of 11 lymphoma cell lines with epigenetic drugs. We identified 233 genes with upregulated expression in treated cell lines and with downregulated expression in B-cell lymphoma patient samples (n = 480) when compared to normal B cells (n = 5). The top 30 genes were further analyzed by methylation specific PCR (MSP) in 18 lymphoma cell lines. Seven of the genes were methylated in more than 70% of the cell lines and were further subjected to quantitative MSP in 37 B-cell lymphoma patient samples (diffuse large B-cell lymphoma (activated B-cell like and germinal center B-cell like subtypes), follicular lymphoma and Burkitt`s lymphoma) and normal B lymphocytes from 10 healthy donors. The promoters of *DSP*, *FZD8*, *KCNH2,* and *PPP1R14A* were methylated in 28%, 67%, 22%, and 78% of the 36 tumor samples, respectively, but not in control samples. Validation using a second series of healthy donor controls (n = 42; normal B cells, peripheral blood mononuclear cells, bone marrow, tonsils and follicular hyperplasia) and fresh-frozen lymphoma biopsies (n = 25), confirmed the results. The DNA methylation biomarker panel consisting of *DSP*, *FZD8*, *KCNH2,* and *PPP1R14A* was positive in 89% (54/61) of all lymphomas. Receiver operating characteristic analysis to determine the discriminative power between lymphoma and healthy control samples showed a c-statistic of 0.96, indicating a possible role for the biomarker panel in monitoring of lymphoma patients.

## Introduction

The transformation of normal cells into cancer cells is a multistep process, involving irreversible changes of the DNA sequence [1]. Non-Hodgkin lymphoma (NHL) is the sixth most common cancer type in the United States with 69 740 new cases per year (2013) [2]. Several of the NHL subtypes are characterized by known chromosome translocations involving immunoglobulin gene loci and different proto-oncogenes, which lead to oncogene activation. Translocations between immunoglobulin genes and *BCL2*, *MYC*, and *BCL1/CYCLIN D1* are found in the majority of follicular Lymphoma (FL), Burkitt`s Lymphoma (BL), and Mantle Cell Lymphoma, respectively [3]–[5]. Interestingly, the *BCL2* translocation can be detected by sensitive methods in the blood of 16–45% of healthy donors [6], indicating that additional aberrations are required for lymphomagenesis.

Aberration in the DNA methylation pattern is known to be a frequent event in cancer. In addition to a global hypomethylation, several gene promoters become hypermethylated in NHL, including well-established tumor suppressor genes such as *CDKN2A* (*p16*) [7], *DAPK* [8], and *CRBP1* [9]. Although the number of methylated genes found in NHL is constantly increasing [10], [11], most studies are focusing on only one NHL type [12]–[15] and so far only a handful studies have examined the putative use of methylation markers as diagnostic or prognostic tools [16]–[18]. This is however of importance since, due to new treatment regimes, established biomarkers for NHL are losing the power of predicting patients outcome. This is underscored by the introduction of rituximab in the treatment of NHL, which diminished the previous prognostic significance of BCL2 [19]. The aim of this study is to identify highly methylated genes across various NHL types. We further analyze the ability of the candidate genes to differentiate lymphoma patients from healthy controls.

## Materials and Methods

### 2.1 Patients and Cell Lines

In the present study we included DNA from 62 fresh-frozen primary diagnostic biopsies of patients diagnosed with B-cell lymphoma (activated B cell like diffuse large B-cell lymphoma (DLBCL ABC); *n* = 18, germinal centre cell like diffuse large B-cell lymphoma (DLBCL GCB); *n* = 17, primary mediastinal B-cell lymphoma (PMBL); *n* = 6, follicular lymphoma (FL); *n = *14 and Burkitt`s lymphoma (BL), *n* = 7) and 52 various healthy donors (CD19^+^-B-cells isolated from buffy coat with CD19^+^ Dynabeads (Invitrogen) as previously described [20]; *n* = 20, follicular hyperplasia samples; *n* = 9, peripheral blood mononuclear cells; *n* = 10, bone marrow, *n* = 3 and tonsils; *n* = 10). The patients included in this study had a median observation time of 36 months, and during this time eight out of 62 patients (13%) died. Additional information about the patients can be found in Table 1. Patients with BL were treated according to an intensified chemotherapy regimen with rituximab (GMALL 2002) and FL patients, if in need of therapy, with rituximab monotherapy, cyclophosphamide, vincristine, predisolone (CVP) plus rituximab or cyclophospamide, doxorubicine, vincristine, prednisolone (CHOP) plus rituximab. DLBCL patients were treated with CHOP-like therapy plus rituximab. All samples analyzed in the present study were collected prior to patient treatment.

**Table 1:** Patient characteristics.

	Test series	Validation series
**Number of patients**	**37**	**25**
** Number of BL**	**7**	**0**
IPI low (0–2)	4	
IPI high (3–5)	3	
Stage 1–2	4	
Stage 3–4	3	
** Number of DLBCL ABC**	**10**	**8**
IPI low (0–2)	4	2
IPI high (3–5)	3	2
Stage 1–2	3	2
Stage 3–4	7	6
** Number of DLBCL GCB**	**10**	**7**
IPI low (0–2)	7	3
IPI high (3–5)	2	1
Stage 1–2	3	3
Stage 3–4	6	4
** Number of FL**	**10**	**4**
FLIPI low (0–2)	5	1
FLIPI high (3–5)	1	1
Stage 1–2	2	1
Stage 3–4	4	3
** Number of PMBL**	**0**	**6**
IPI low (0–2)		2
IPI high (3–5)		0
Stage 1–2		4
Stage 3–4		2
**Male/female quotient**	**3,1**	**0,9**
**Median age, year (range)**	**61 (34–73)**	**55 (29–81)**

The international prognostic index (IPI) and follicular lymphoma IPI (FLIPI) status or stage could not be obtained from all patients. Abbreviations: Burkitt`s lymphoma (BL), diffuse large B-cell lymphoma (DLBCL) activated B-cell type (ABC), germinal center B-cell type (GCB), follicular lymphoma (FL) and primary mediastinal B-cell lymphoma (PMBL).

Eighteen B-cell lymphoma cell lines were examined: BL: BL41 (purchased from DSMZ, Germany), Namwalwa (ATCC), Raji and Ramos (DSMZ); DLBCL ABC: HLY-1 [21], OciLy3 [22], OciLy10 [22], and U2932 (DSMZ); DLBCL GCB: NUDHL1, OciLy2, OciLy7, OciLy19, SUDHL4, and SUDHL10 (L. Staudt), SUDHL6 (DSMZ); and FL: K422, SC-1 and ROS50 (DSMZ). OciLy2, -3, -7, -10, and -19 were cultured in IMDM medium (Invitrogen) supplemented with 20% human plasma (SeraCare Life Sciences, Inc.; California, USA), 55 µM β-mercaptoethanol (Invitrogen), 100 Units/ml penicillin and 0.1 mg/ml streptomycin (PAA Laboratories) at 37°C with 5% CO_2_. The remaining lymphoma cell lines were cultured in RPMI 1640 (PAA Laboratories, Austria), supplemented with 10% fetal calf serum (PAA Laboratories), 100 Units/ml penicillin and 0.1 mg/ml streptomycin (PAA Laboratories) at 37°C with 5% CO_2_. All cell lines have been authenticated by STR-loci analysis, which has been compared to the database of DMZG. The STR results of non-commercially available cell lines will be provided on request.

### 2.2 Ethical Statement

The study has been performed in accordance with the Declaration of Helsinki and is approved by the Regional Committees for Medical and Health Research Ethics, Region Eastern Norway (S-05145). An informed consent has been signed by all patients who have been included in this study.

### 2.3 Nucleic Acid Isolation

DNA and total RNA from cell lines and CD19^+^-B-cells were isolated using the AllPrep DNA/RNA/protein Kit from Qiagen. Concentrations were measured using the ND-1000 NanoDrop (Thermo Scientific). RNA quality was measured with the 2100 Bioanalyzer (Agilent Technologies).

### 2.4 Epigenetic Drug Treatment of Lymphoma Cell Lines

Eleven B-cell lymphoma cell lines (BL: Raji, BL41, Ramos; DLBCL ABC: HLY-1, OciLy3, OciLy10; DLBCL GC: SUDHL4, SUDHL6; and FL: K422, SC-1, ROS50) were treated with a combination of the demethylating reagent 5-aza-2′deoxycytidine (aza; 1 µM for 72 h) and the histone deacetylase inhibitor trichostatin A (TSA; 0.5 µM added the last 12 h). The same cell lines, cultured in parallel without treatment, were used as a control.

### 2.5 Gene Expression Microarray Analysis

Epigenetically drug treated cell lines and their untreated counterparts were analyzed with the Applied Biosystems Human Genome Survey Microarray following manufacturer’s protocol. In brief, 1.5 µg of total RNA was labeled using the Chemiluminiescent RT-IVT Labeling Kit from Applied Biosystems. Hybridization was performed at 55°C for 16 h using 10 µg of the labeled cRNA. Chemiluminescence detection and image analysis were performed using Applied Biosystems Chemiluminescence Detection Kit and Applied Biosystems 1700 Chemiluminescent Microarray Analyzer according to the manufacturer’s protocol. Post-processing and normalization was done with the R-script “ABarray” and Bioconductor. Only array elements that were at least 2-fold up-regulated after the epigenetic drug treatment in at least 6 out of the 11 analyzed cell lines, were considered to be candidate genes for methylation in B-cell lymphoma cell lines. Raw data have been deposited in the Gene Expression Omnibus (GEO) public repository for microarray data (accession number GSE46064).

Gene expression data from 480 B-cell lymphomas (BL *n = *24, GSE 4732 [23]; DLBCL ABC *n = *168, GEO accession number GSE10846 [24]; DLBCL GC *n = *97, GEO accession number GSE10846 [24]; FL *n = *191, unpublished) were accessible for the project from the Leukemia Lymphoma Molecular Profiling Project (LLMPP). To compare the tumor gene expression to healthy donors, RNA from CD19^+^-B-cells (*n = *5) was analyzed on the same Affymetrix HG-U133 Plus 2.0 arrays and were normalized with the same protocol as the tumor samples in a LLMPP facility (GEO accession number GSE46062).

### 2.6 Experimental Strategy for Identifying Methylated Candidate Genes

**Figure 1:**
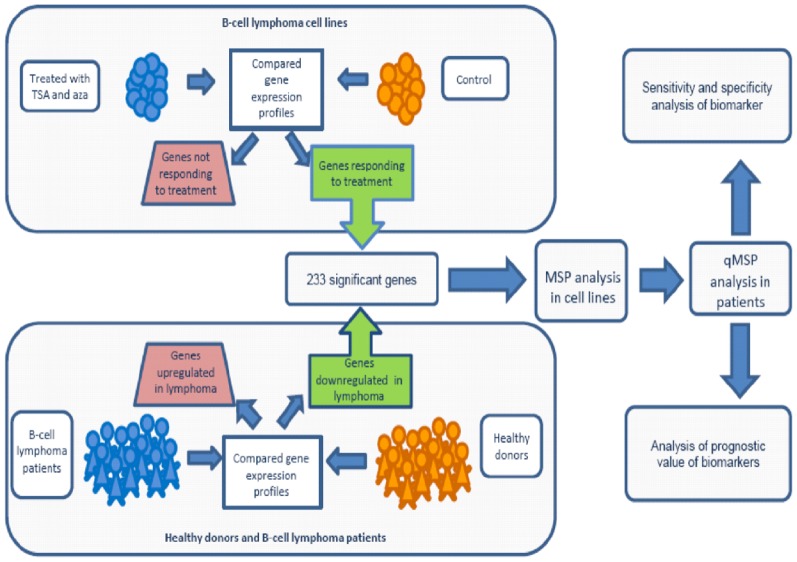
Experimental setup. Gene expression profiling was performed on 11 B-cell lymphoma cell lines, with and without epigenetic drug treatment. Genes responding to the epigenetic drugs were further analyzed in B-cell lymphoma patients (*n* = 480) compared to B cells from healthy donors (*n* = 5) to see if they were downregulated. These criteria were met by 233 genes. The top 30 genes were selected for Methylation-specific-PCR (MSP) analysis in 18 B-cell lymphoma cell lines. Genes with a methylation frequency above 70% in cell lines were further validated by quantitative MSP (qMSP) in B-cell lymphoma patients (*n* = 37). The biomarker potential (both for detection and prognostication) of the candidates was subsequently evaluated.

To increase the likelihood of selecting appropriate candidates for DNA methylation in B-cell lymphomas, we used a multistep strategy focusing on genes that in addition to being upregulated by epigenetic drug treatment in cell lines were also downregulated in lymphomas compared to normal CD19^+^-B-cells. The candidate genes were subject to further analyses in cancer cell lines (MSP) and finally in patient material (qMSP) (Figure 1).

### 2.7 Methylation Specific Polymerase Chain Reaction (MSP)

All DNA methylation candidates from the array approach mentioned above were analyzed using the RefSeqs from the UCSC Genome browser database (http://genome.ucsc.edu/) and default settings in the CpG Island Searcher Software [25] in order to see if they had a CpG island present in their promoter. The input sequence included 1000 bp upstream and 500 bp downstream of the transcription start site.

Genes containing a promoter CpG-Island were analyzed by MSP in all cell lines (*n = *18) and in CD19^+^-B-cells. Primers were designed using the Methyl Primer Express 1.0 Applied Biosystems, their sequences are provided in Table S1. DNA from normal blood and *in vitro* SssI methyltransferase (New England Biolabs Inc.) treated DNA (Human placenta DNA (Sigma)), was used as an unmethylated and methylated positive control, respectively, and dH_2_O replacing the bisulfite template was the negative control in both reactions.

For each sample, 1.3 µg DNA was bisulfite treated with the EpiTect bisulfite kit (Qiagen), according to the manufacturer’s protocol. For the MSP reaction the HotStarTaq polymerase (0.6 units) was used along with 10x PCR buffer containing MgCl_2_ (all Qiagen), dNTP mix (10 nM each; Roche), and 20 pmol of each primer (Eurofins MWG operon, Germany). Approximately 32.5 ng bisulfite-converted DNA was used as template and the total volume of the PCR reactions was 25 µl. The following PCR program was used: 15 min at 95°C to activate the enzyme; followed by 35 cycles: 95°C for 30 sec (denaturation), annealing for 30 sec, and 72°C for 30 sec (elongation). A final elongation at 72°C for 7 min completed the PCR reaction. PCR products were loaded on a 2% agarose gel, stained with SYBR Safe (Invitrogen), and visualized by UV irradiation using a Geldoc (Biorad). For all samples and all genes, two independent PCR reactions were performed.

### 2.8 Bisulfite Sequencing

Bisulfite sequencing primers were designed using Methyl Primer Express 1.0 (Applied Biosystems) to flank the MSP primer binding sites in the respective gene promoter. Primer sequences are provided in Table S1. *KCNH2* was not sequenced since the high CpG density of the promoter region in question made it challenging to amplify the unmethylated and methylated alleles equally efficient.

For the initial amplification the same PCR conditions as for the MSP was applied. PCR products were cleaned from excess primer and nucleotides with ExoSAP-IT (GE Healthcare) following the manufactures instructions. The purified products were sequenced using the Big Dye sequencing kit 1.1 in an ABI Prism 3700 Genetic Analyzer (Applied Biosystems). The approximate amount of methyl cytosine of each CpG site was calculated by comparing the peak height of the cytosine signal with the sum of the cytosine and thymine peak height signals. Unmethylated CpG sites included ratios between 0 and 0.20, partially methylated included ratios from 0.21 to 0.80, and a ratio from 0.81 to 1.0 was considered to be fully methylated.

### 2.9 Quantitative Methylation-specific Polymerase Chain Reaction (qMSP)

Primers and probes for qMSP were designed with Applied Biosystems Primer Express 3.0 Software to anneal to bisulfite treated and fully methylated DNA (sequences are provided in Table S1). In a 20 µl reaction, approximately 32.5 ng bisulfite treated DNA was used as template in addition to 10 µl 2xTaqMan Universal PCR Master Mix No AmpErase UNG (Applied Biosystems), 0,9 µM of forward and reverse primer and 0,2 µM probe. The PCR program started with an incubation step at 95°C for 10 min, followed by 45 cycles of 95°C for 15 sec and 60°C for 1 min. The samples were run in triplicates on a ABI Prism 7900 HT Sequence detection system and analyzed with the sequence detector system 2.3 (Applied Biosystems). The analyzed genes were normalized for DNA input using ALU-C4 as a reference gene [26]. A standard curve of bisulfite treated universal methylated DNA (Chemicon, Millipore) was used to determine the quantity of methylated DNA in each sample.

For all samples, amplification after cycle 35 was censored. We calculated the percent of methylated reference (PMR) by using the median GENE:ALU ratio of a sample and divided it by the median GENE:ALU ratio of the positive control (CpGenome Universally Methylated DNA) and multiplied it by 100. The highest PMR value across the healthy controls was used as a threshold for scoring samples as methylation positive (Table S2).

## Results

### 3.1 Identification of Methylated Candidate Genes in B-cell Lymphoma

An overview of the experimental design as well as how candidate genes have been selected for subsequent DNA promoter methylation analysis is provided in Figure 1. The epigenetic drug treatment of B-cell lymphoma cell lines (BL: Raji, BL41, Ramos; DLBCL ABC: HLY-1, OciLy3, OciLy10; DLBCL GC: SUDHL4, SUDHL6; FL: K422, SC-1, ROS50) with 5-aza-2′deoxycytidine (aza) and Trichostatin A (TSA) and a subsequent genome-wide expression analysis revealed that 2027 array elements were upregulated a minimum of two fold in at least 6 out of the 11 analyzed cell lines.

**Figure 2:**
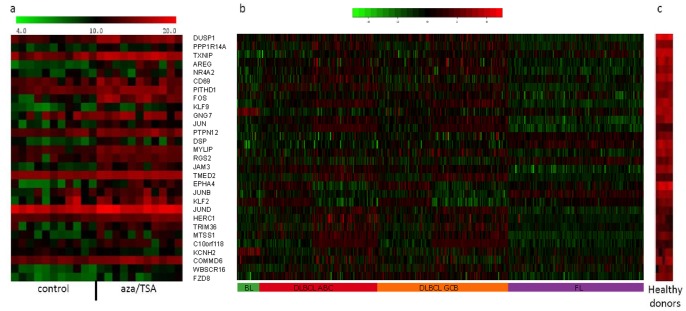
Gene expression profiles of 30 candidate genes. Gene expression profiles of 11 B-cell lymphoma cell lines treated (aza/TSA) and untreated (a), 480 B-cell lymphomas (b) and CD19^+^-B-cells from five healthy donors (c) for the 30 candidate genes for DNA methylation. Genes have been sorted according to the level of downregulation in patients compared to healthy controls.

From a dataset of 480 B-cell lymphomas and 5 normal peripherial blood CD19^+^-B-cells, we identified 5736 downregulated array elements. Downregulated elements included all elements with a lower median expression value across all lymphoma samples compared to the median expression value of the controls. The overlap between the two microarray dataset analyses (the 2027 upregulated array elements from the cell line approach and the 5736 downregulated array elements from gene expression microarray analysis of clinical samples) consisted of 233 genes (Figure 1). This list was finally ranked according to the degree of downregulation in lymphoma samples. The top 30 genes were subjected to downstream analysis (Figure 2 and Tables 2 and S3).

**Table 2:** Methylation status of candidate genes in 18 B-cell lymphoma cell lines.

	BL41	HLY-1	K422	Namwalwa	NUDHL1	Ocily-10	Ocily-19	Ocily-2	Ocily-3	Ocily-7	Raji	Ramos	Ros-50	SC-1	Sudhl10	Sudhl4	Sudhl6	U2932	Methylation (%)
**PPP1R14A**	M	U/M	M	M	M	U/M	M	M	M	M	M	M	M	M	M	M	M	U/M	**100**
**DSP**	U/M	M	M	M	U/M	U/M	U/M	U/M	M	U/M	M	U/M	U/M	U/M	U/M	M	U/M	U	**94**
**FZD8**	M	M	M	U/M	M	M	M	M	U	M	M	M	U/M	M	M	M	M	U	**89**
**NR4A2**	U/M	U/M	U/M	U/M	U/M	U	U/M	U/M	U	U/M	M	M	U/M	U/M	U/M	U/M	U/M	U/M	**89**
**MTSS1**	U/M	U/M	U/M	U/M	U/M	U	U/M	U/M	U	U/M	U/M	U/M	U/M	U	U/M	U/M	U/M	U/M	**83**
**KCNH2**	M	U/M	U/M	U/M	U	U/M	M	U/M	U/M	U	U/M	M	U/M	U/M	U	U/M	U/M	U	**78**
**KLF9**	U/M	U	U/M	U/M	U/M	U/M	U	U/M	U/M	U/M	U/M	U/M	U	U	U/M	U/M	U/M	U	**72**
PTPN12	U/M	M	U	U	U	U/M	U	U/M	U/M	U/M	M	U/M	U	U/M	U/M	U	U	U	56
JAM3	U	U/M	U	U/M	U	U/M	U	U/M	M	U/M	M	U/M	U/M	U	U	U/M	U	U	56
TRIM36	U/M	U/M	M	U/M	U/M	U	U/M	U	U	U/M	M	U/M	U	U	U	U	U	U	50
MYLIP	U	U	M	U/M	U/M	U	U	U/M	U/M	U	U	M	U/M	U	M	U/M	U	U	50
AREG	U	U	U	U/M	U	U	U	U/M	U	U/M	U/M	U/M	U	U	U	U	U	U	28
KLF2	U	U	U	U	U	U	U	U	U/M	M	M	M	U	U	U	U/M	U	U	28
EPHA4	U	U/M	U	U	U/M	U	U	U	U	U	U/M	U/M	U	U	U	U	U/M	U	28
GNG7	U	U	U	U	U	M	U	U	M	U	U	U	U	U	U	U	U	U/M	17
RGS2	U	U	U	U	U	U	U/M	U	U	U	U/M	U	U	U	U	U	U	U	11
TMED	U	U	U	U	U	U	U	U	U/M	U	U	U/M	U	U	U	U	U	U	11
C10orf118	U	U	U	U	U	U	U	U	U	U	U/M	U	U	U	U	U	U	U	6
COMMD6	U	U	U	U	U	U	U	U	U	U	U	U	U	U	U	U	U	U	0
DUSP1	U	U	U	U	U	U	U	U	U	U	U	U	U	U	U	U	U	U	0
FOS	U	U	U	U	U	U	U	U	U	U	U	U	U	U	U	U	U	U	0
HERC1	U	U	U	U	U	U	U	U	U	U	U	U	U	U	U	U	U	U	0
JUN	U	U	U	U	U	U	U	U	U	U	U	U	U	U	U	U	U	U	0
JUNB	U	U	U	U	U	U	U	U	U	U	U	U	U	U	U	U	U	U	0
JUND	U	U	U	U	U	U	U	U	U	U	U	U	U	U	U	U	U	U	0
PITHD1	U	U	U	U	U	U	U	U	U	U	U	U	U	U	U	U	U	U	0
TXNIP	U	U	U	U	U	U	U	U	U	U	U	U	U	U	U	U	U	U	0
WBSCR16	U	U	U	U	U	U	U	U	U	U	U	U	U	U	U	U	U	U	0

MSP analysis of 28 gene promoters in 18 B-cell lymphoma cell lines. Abbreviations: M, methylated promoter region; U, unmethylated promoter region; U/M partially methylated promoter region. Genes are sorted according to their methylation frequency in the cell lines.

### 3.2 Analysis of Methylation Candidates in Cell Lines Using Methylation Specific PCR (MSP)

The promoter region of the top 30 candidate genes were first analyzed for the presence of CpG-islands. With the exception of *CD69* and *SLC2A3*, all candidate genes had a promoter CpG-island. The majority of the analyzed genes displayed variable promoter methylation frequencies among the 18 B-cell lymphoma cell lines (Table 2 and Figure S1), and were unmethylated in normal B cells. Seven genes (*DSP*, *FZD8*, *KCNH2*, *KLF9*, *MTSS1*, *NR4A2,* and *PPP1R14A*) were individually methylated in more than 70% of the cell lines, and were further subjected to quantitative methylation analysis in patient and normal samples.

### 3.3 Bisulfite Sequencing of Promoter CpG Sites

The methylation status of the individual CpG sites in parts of the promoter of the candidates (*DSP*, *FZD8*, *KLF9*, *MTSS1,* and *NR4A2*) was analyzed by bisulfite sequencing in order to guide the design of qMSP assays so that the primer and probe binding sites include frequently methylated CpG sites. For *PPP1R14A* we used a qMSP assay previously designed by us. The bisulfite sequencing results are visualized in Figure S2, along with the result of the individual MSP analyses and the location of primers and probes of the qMSP assays. Furthermore, the bisulfite sequencing revealed a high bisulfite conversion rate, since all non-CG cytosines were converted to thymines.

### 3.4 Quantitative Methylation Analysis of Patients and Healthy Donors

**Figure 3:**
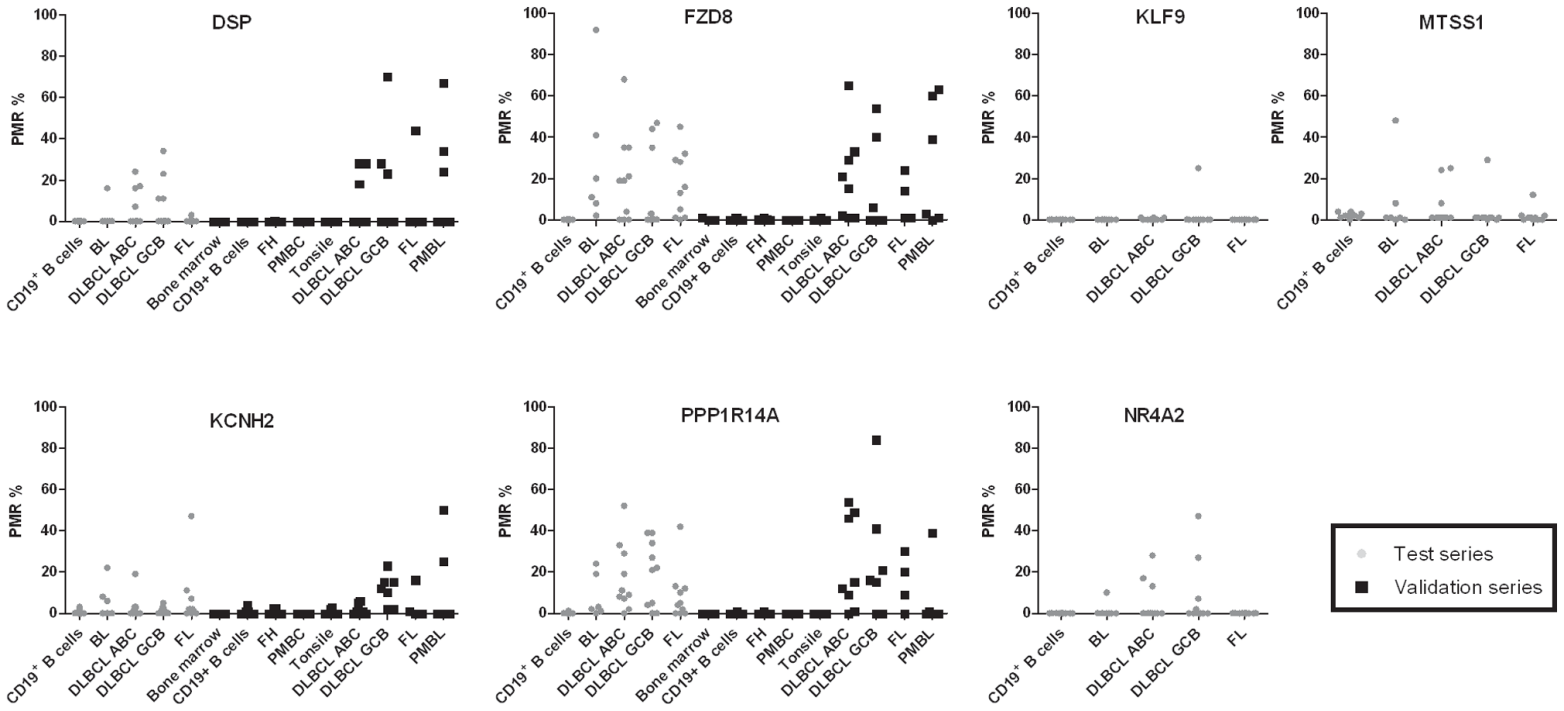
Percent promoter methylation of the analyzed genes in the test and validation series. Each point represents one patient tumor sample. Abbreviations: BL, Burkitt`s lymphoma; DLBCL ABC, activated B-cell like diffuse large B-cell lymphoma; DLBCL GCB, germinal centre B-cell like diffuse large B-cell lymphoma; FL, follicular lymphoma; PBMC, peripheral blood mononuclear cells; PMBL, primary mediastinal B-cell lymphoma PMR, percent methylated reference.

By qMSP we analyzed the promoter methylation status of *KLF9*, *MTSS1*, *NR4A2, KCNH2, DSP, FZD8,* and *PPP1R14A* in 37 NHL patients and CD19^+^-B-cells from 10 healthy donors. The overall promoter methylation across all analyzed NHL types was 11% (4/37), 19% (7/37), 22% (8/37), 22% (8/36), 28% (10/36), 67% (24/36), and 78% (28/36), respectively (Figure 3 and Table 3). All genes were unmethylated (PMR = 0) in CD19^+^-B-cells from healthy donors.

**Table 3:** Methylation frequencies and range of PMR values (brackets) assessed by qMSP in the clinical test and validation sets.

Test series	BL	DLBCL ABC	DLBCL GCB	FL	NHL
***DSP***	1/6 (17%)	4/10 (40%)	4/10 (40%)	1/10 (10%)	10/36 (28%)
	[16]	[7]–[24]	[11]–[34]	[3]	[3]–[34]
***FZD8***	6/6 (100%)	7/10 (70%)	4/10 (40%)	7/10 (70%)	24/36 (67%)
	[2–92]	[4–68]	[3–47]	[5]–[45]	[2–92]
***KCNH2***	3/6 (50%)	1/10 (10%)	1/10 (10%)	3/10 (30%)	8/36 (22%)
	[6]–[22]	[19]	[5]	[7–47]	[5–47]
*KLF9*	0/7 (0%)	3/10 (30%)	1/10 (10%)	0/10 (0%)	4/37 (11%)
	[0]	[1]	[25]	[0]	[1]–[25]
*MTSS1*	2/7 (29%)	3/10 (30%)	1/10 (10%)	1/10 (10%)	7/37 (19%)
	[8–48]	[8]–[25]	[29]	[12]	[8–48]
*NR4A2*	1/7 (14%)	3/10 (30%)	4/10 (40%)	0/10 (0%)	8/37 (22%)
	[10]	[13]–[28]	[2–47]	[0]	[2–47]
***PPP1R14A***	4/6 (67%)	9/10 (90%)	8/10 (80%)	7/10 (70%)	28/36 (78%)
	[2]–[24]	[2–52]	[4]–[39]	[2]–[42]	[2–52]
***combined biomarker panel***	**(6/6) 100%**	**(10/10) 100%**	**(8/10) 80%**	**(10/10) 100%**	**(34/36) 94%**
**Validation series**	**DLBCL ABC**	**DLBCL GCB**	**FL**	**PMBL**	**NHL**
***DSP***	3/8 (38%)	3/7 (43%)	1/4 (25%)	3/6 (50%)	10/25 (40%)
	[18]–[28]	[23–70]	[44]	[24–67]	[18–70]
***FZD8***	6/8 (75%)	3/7 (43%)	2/4 (50%)	4/6 (67%)	15/25 (60%)
	[2–65]	[6–54]	[14]–[24]	[3–63]	[2–65]
***KCNH2***	2/8 (25%)	5/7 (71%)	1/4 (25%)	2/6 (33%)	10/25 (40%)
	[5]–[6]	[10]–[23]	[16]	[25–50]	[5–50]
***PPP1R14A***	6/8 (75%)	5/7 (71%)	3/4 (75%)	1/6 (17%)	15/25 (60%)
	[9–54]	[15–84]	[9]–[30]	[39]	[9–84]
***combined biomarker panel***	**(7/8) 88%**	**(6/7) 86%**	**(3/4) 75%**	**(4/6) 67%**	**(20/25) 80%**

The various healthy controls were unmethylated in all analyzed markers. Genes in bold (*DSP*, *FZD8*, *KCNH2*, *PPP1R14A*) are included in the combined biomarker panel. Abbreviations: Burkitt`s lymphoma (BL), diffuse large B-cell lymphoma (DLBCL) activated B-cell type (ABC), germinal center B-cell type (GCB), follicular lymphoma (FL), primary mediastinal B-cell lymphoma (PMBL) and non-Hodgkin lymphoma (NHL).

The four best-performing candidates from the test series (*DSP, FZD8*, *KCNH2,* and *PPP1R14A*) were further subjected to promoter methylation analyses in a validation series, which included additional NHL samples (*n = *25) and healthy controls (bone marrow, tonsils, peripheral blood mononuclear cells and follicular hyperplasia samples, *n = *42). We applied the threshold for scoring methylation-positive samples from the test series and found the following individual promoter-methylation frequencies of *DSP*, *FZD8*, *KCNH2,* and *PPP1R14A* across the different NHL types in the validation series: 40% (10/25), 60% (15/25), 40% (10/25), and 60% (15/25), respectively (Figure 3 and Table 3). All genes were unmethylated in the analyzed healthy controls (100% specificity). Across the test and validation series 54 of the 61 successfully analyzed patients had methylation of one or more genes in the panel (*DSP, FZD8, KCNH2*, and *PPP1R14A*) reaching a sensitivity of 89% and a specificity of 100%.

### 3.5 Receiver Operating Characteristics Curves

**Figure 4:**
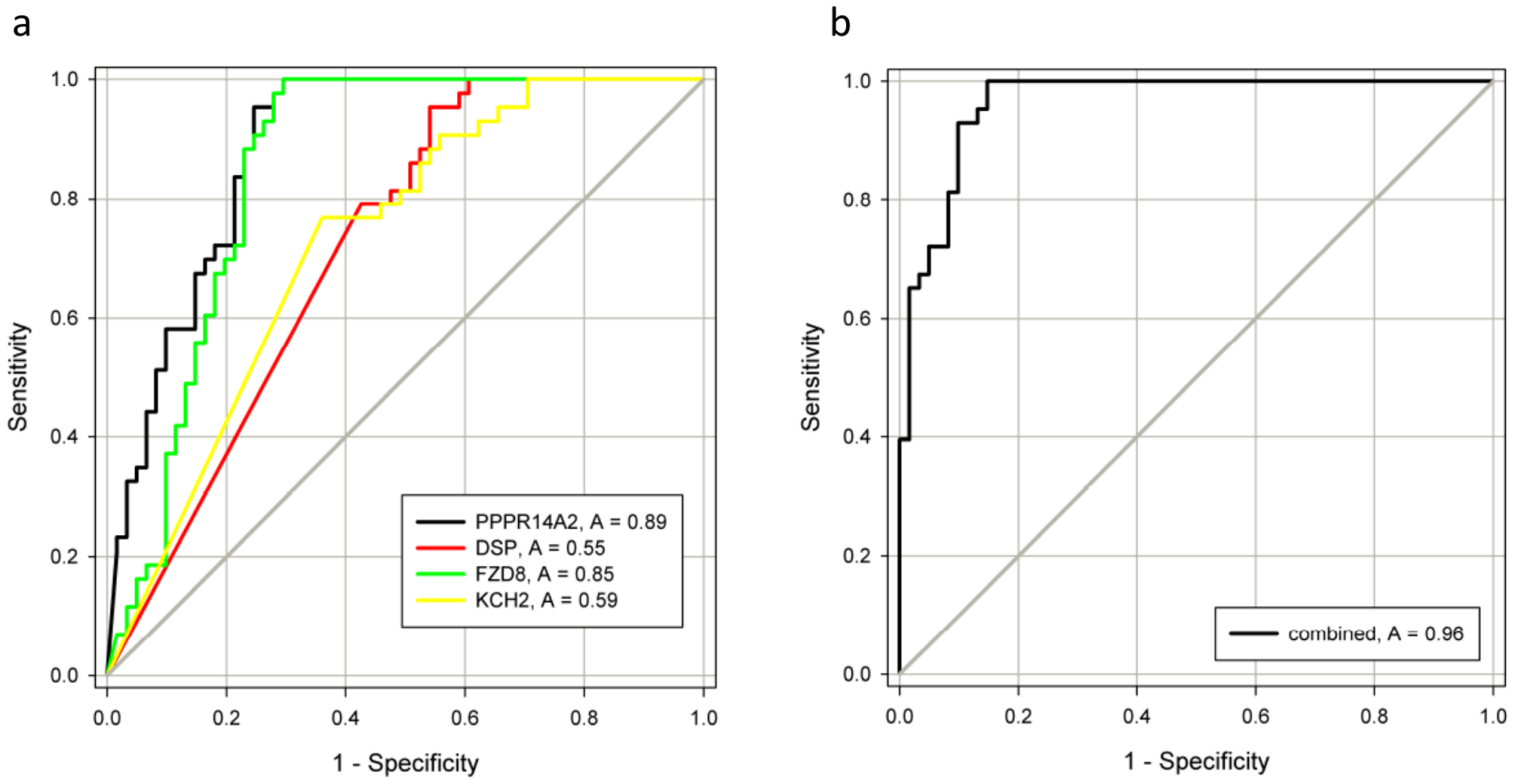
Receiver Operating Characteristics (ROC) curves for individual and combined markers in lymphoma patients versus healthy donors. The area under the ROC curve (AUC) represents how accurate the individual and combined biomarkers can discriminate between lymphomas and normal controls. A) Individual genes. B) The combined gene panel.

The PMR values from the qMSP analysis were used to generate receiver operating characteristics (ROC) curves. Due to low methylation frequencies, the genes *KLF9*, *MTSS1,* and *NR4A2* were only analyzed in the test series, resulting in an area under the ROC curve (AUC) of 0.17, 0.34, and 0.44, respectively. The remaining genes *DSP*, *FZD8*, *KCNH2,* and *PPP1R14A* were analyzed in both the test and validation series and showed an individual AUC of 0.55, 0.85, 0.59 and 0.89, respectively across both series (Figure 4a). The combined panel of *DSP*, *FZD8*, *KCNH2,* and *PPP1R14A*, based on the sum of the PMR values, generated an AUC of 0.96 (Figure 4b).

## Discussion

We have previously established a stepwise experimental approach to identify novel methylated biomarkers in colorectal cancer (CRC) [27]–[29]. In the present study, we used a similar approach, and combined gene expression data from a large panel of lymphoma cell lines treated with a demethylating agent with the gene expression in NHL patient biopsies and normal peripheral blood B cells from healthy controls. Using this approach we successfully identified novel methylated genes in NHL. Four genes, *DSP*, *FZD8*, *KCNH2,* and *PPP1R14A* were frequently methylated across the major NHL types. The combination of these four genes could successfully discriminate NHL samples (89%) from healthy controls (normal B lymphocytes isolated from blood, bone marrow samples, peripheral blood mononuclear cells, tonsils and follicular hyperplasia samples) as shown by Receiver Operating Characteristic analysis with a c-statistic (area under the curve) of 0.96.

Cancers generally harbor several hypermethylated promoter regions [30], and different methodological approaches are expected to identify various subsets of these. In spite of the numerous methylated genes identified so far only a limited number have high enough performance to qualify as biomarkers. In this study, we identified several methylated genes in lymphoma and/or lymphoma cell lines. Interestingly several of these have previously been shown to be altered in other types of cancers. *MTSS1* and *DSP* are known tumor suppressor genes and are together with *PPP1R14A,* also methylated in lung-, colorectal- and gastric cancer [27], [31]–[33]. However, this is, to the best of our knowledge, the first time *DSP*, *FZD8*, *KCNH2, MTSS1,* and *PPP1R14A* have been reported to be methylated in lymphoma. No cancer-relevant role has so far been reported for the *PPP1R14A* gene, which encodes a protein phosphatase, and is involved in regulating the contraction in smooth-muscle tissue. The gene *KCNH2*, also known as *hERG1*, encodes a potassium channel and has been shown to regulate cell proliferation, apoptosis, cell invasion, and angiogenesis by modulating several biochemical pathways [34]. These effects are mediated by *KCNH2* recruitment into the plasma membrane as well as by an interaction with integrins and growth factors [35]. Interestingly, the gene with the second highest methylation frequency in the present study, Frizzled family receptor 8 (*FZD8)*, is involved in the Wnt signaling pathway, which is frequently altered among several cancer types, including leukemia and CRC [36], [37]. DSP has also been shown to be involved in the Wnt signaling pathway [32] and has been shown to be expressed in gastro-intestinal follicular lymphoma, a subtype with a favorable prognosis [38]. The prognostic value of all genes in general and *DSP* in particular was tested in our sample cohort. We separated the lymphoma patients into two groups according to methylation status of the gene promoter. However, no prognostic differences were found (data not shown). This is not surprising, considering the small sample series which represents a limitation to the present study. A second limitation is the use of CD19^+^-B-cells as normal controls. Recently, it has been shown that epigenetic heterogeneity is initiated in normal germinal centre B-cells and increases markedly with disease aggressiveness in germinal centre-derived lymphomas [39], [40]. Normal flow-sorted germinal centre B cells could have been an alternative control and would undoubtedly have led to a different candidate gene list. However, CD19^+^ peripheral blood B cells are easy to access and are therefore widely used [41]. Furthermore, we wanted to identify genes which were methylated in lymphoma but not in normal blood or bone marrow cells, to be able to examine the potential for detecting small amounts of neoplastic B cells in blood or bone marrow in future studies.

In contrast to most other studies [14], [42], we have identified several genes which were methylated across NHL types. This includes the primary mediastinal B-cell lymphomas which in general were less frequently methylated for the combined biomarker panel compared with the other NHL types. Interestingly, these lymphomas have a gene expression pattern that resembles that of Hodkin’s lymphoma. Considering the significant molecular, phenotypical and clinical differences between the various lymphoma types, the “universal DNA methylation markers” identified here could potentially be useful for the screening or monitoring of NHL patients. However, this would require a standardized and sensitive test for the analysis of blood or bone marrow samples. A proof of principle has been shown in a study by Tao et al., where the *DLC1* methylation status was analyzed in serum from patients with Hodgkin’s lymphoma [18]. Furthermore the usage of DNA methylation as a biomarker has been shown in sputum for lung cancer, urine sediments for bladder and prostate cancer and stool and plasma for colorectal cancer [43]–[46].

In conclusion, we have discovered novel methylated target genes with high sensitivity and specificity across several lymphoma types.

## Supporting Information

Figure S1 **Representative results from methylation-specific PCR analysis of cancer cell lines.** Representative examples of methylation status of *KCNH2, PPP1R14A, FZD8*, and *NR4A2* in five B-cell lymphoma cell lines (BL41, Ocily7, K422, Ramos, and SUDHL10). NB and IVD are positive controls for the unmethylated and methylated reaction, respectively. A visible PCR product in Lanes U indicates the presence of unmethylated alleles whereas a PCR product in Lanes M indicates the presence of methylated alleles. Abbreviations: IVD, *in vitro* methylated DNA; L, ladder: M, lane for methylated MSP product; NB, normal blood; U, lane for unmethylated MSP product.
(PDF)

Figure S2 **Bisulfite sequencing results of B-cell lymphoma cell lines.** For each gene, the upper panel includes a representative part of the bisulfite sequencing electropherogram. Beneath is a schematic presentation of the individual CpG sites (vertical bars) in the area of transcription start amplified by the bisulfite sequencing primers. The qMSP and MSP primer and probe binding sites are indicated (arrows and straight line, respectively) along with the transcription start site (represented by +1). Twelve cell lines have been sequenced. Each sequenced CpG site is represented by a circle. The color white, gray and black indicates an unmethylated, partial methylated and fully methylated site, respectively. The column in the right side of each panel lists the methylation status for individual samples from MSP analyses. Abbreviations: U, unmethylated; M, methylated and U/M partially methylated.
(PDF)

Table S1 **PCR primer and probe sequences for methylation specific PCR, bisulfite sequencing, and quantitative methylation specific PCR.** Abbreviations: BSP, bisulfite sequencing primers; M, methylated reaction; MSP, methylation specific PCR; qMSP, quantitative methylation specific PCR; U, unmethylated reaction.
(PDF)

Table S2 **Threshold for calling samples as methylated or unmethylated based on the PMR value.** For each gene, samples with PMR values equal to and greater than the indicated threshold were scored as methylated. Samples with lower values were scored as unmethylated. Abbreviations: PMR, percent methylated reference.
(PDF)

Table S3 **Top 30 identified candidate genes for DNA methylation in NHL.** Gene symbol, full gene name and chromosomal location are listed. All genes with CpG islands were subjected to MSP analysis in B-cell lymphoma cell lines. Genes in bold were further validated in clinical patient material by qMSP analysis.
(PDF)
