# Supplementary figures and images for: Identification of Highly Methylated Genes across Various Types of B-Cell Non-Hodgkin Lymphoma
Source: PLoS One. 2013 Nov 19;8(11):e79602. doi: 10.1371/journal.pone.0079602 (PMC3834187; doi:10.1371/journal.pone.0079602)

Figure S1

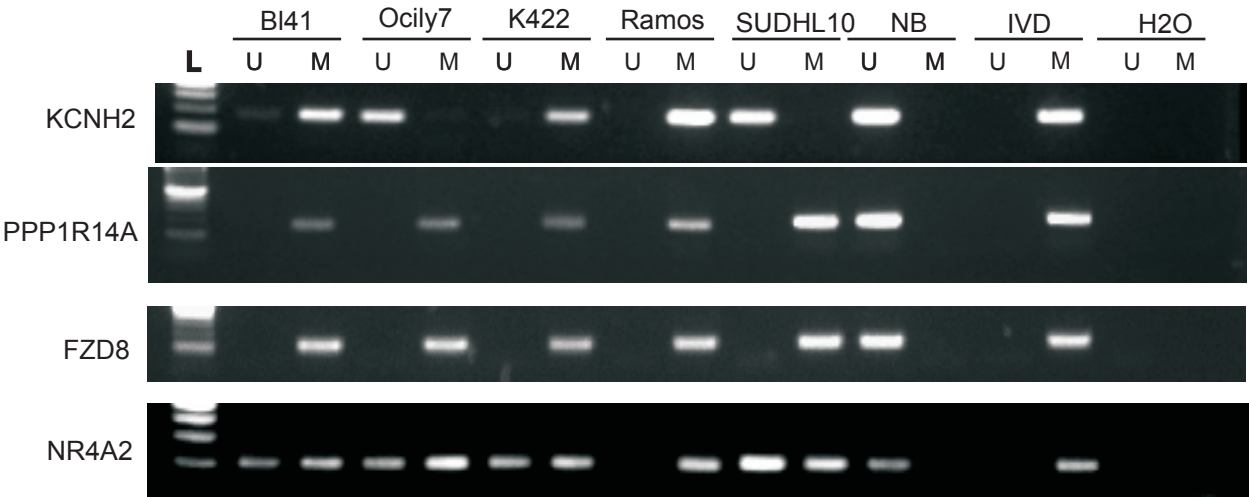

Supplement: Figure S1 — Representative results from methylation-specific PCR analysis of cancer cell lines. Representative examples of methylation status of KCNH2, PPP1R14A, FZD8, and NR4A2 in five B-cell lymphoma cell lines (BL41, Ocily7, K422, Ramos, and SUDHL10). NB and IVD are positive controls for the unmethylated and methylated reaction, respectively. A visible PCR product in Lanes U indicates the presence of unmethylated alleles whereas a PCR product in Lanes M indicates the presence of methylated alleles. Abbreviations: IVD, in vitro methylated DNA; L, ladder: M, lane for methylated MSP product; NB, normal blood; U, lane for unmethylated MSP product. (PDF) [file pone.0079602.s001.pdf]

Figure S2

A. DSP

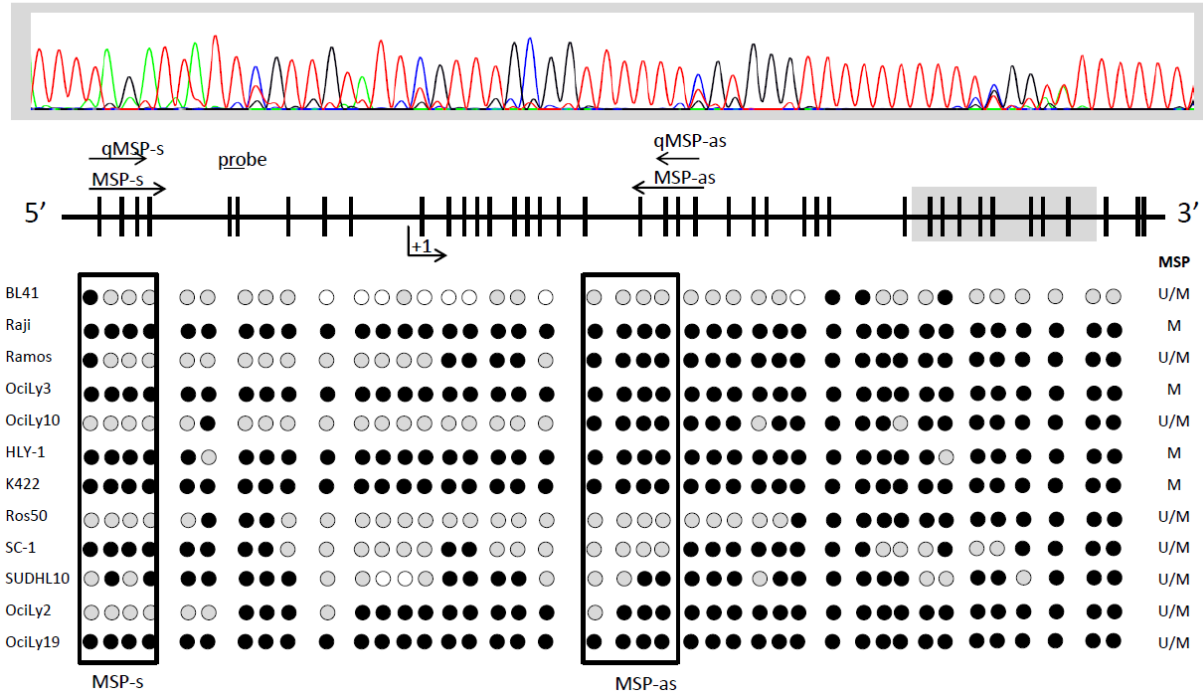

B. FZD8

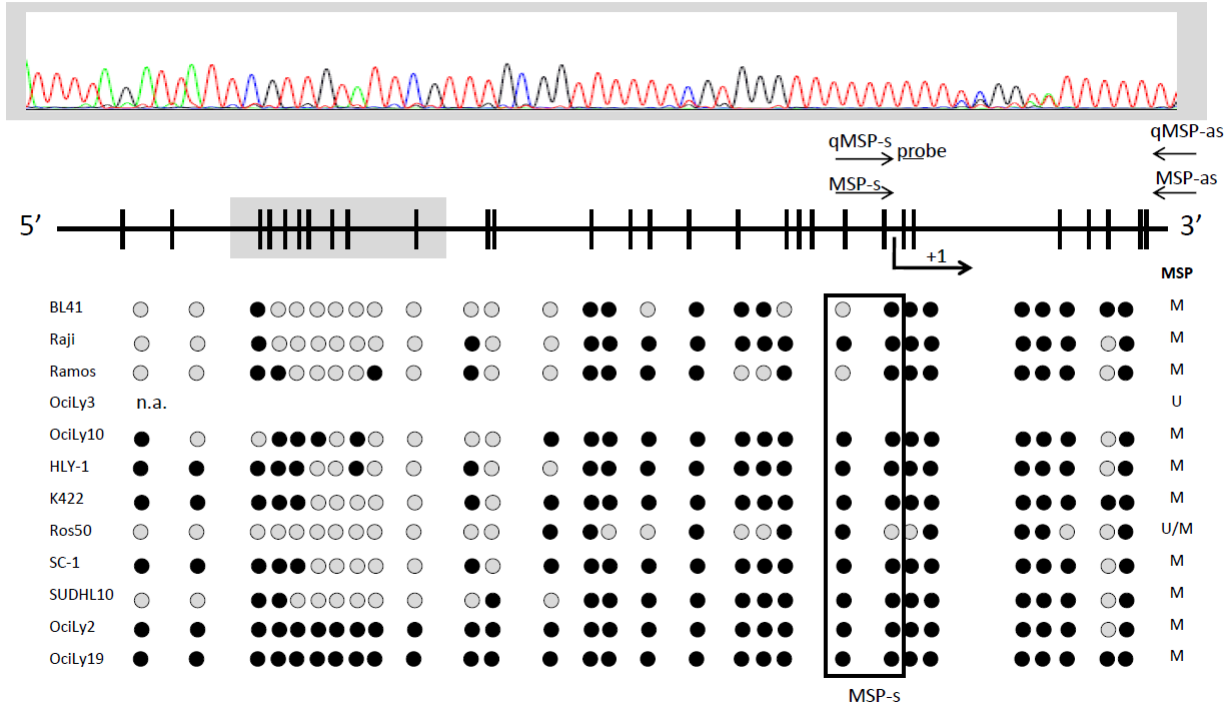

## KLF9

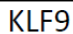

## MTSS1

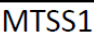

E. **NR4A2**

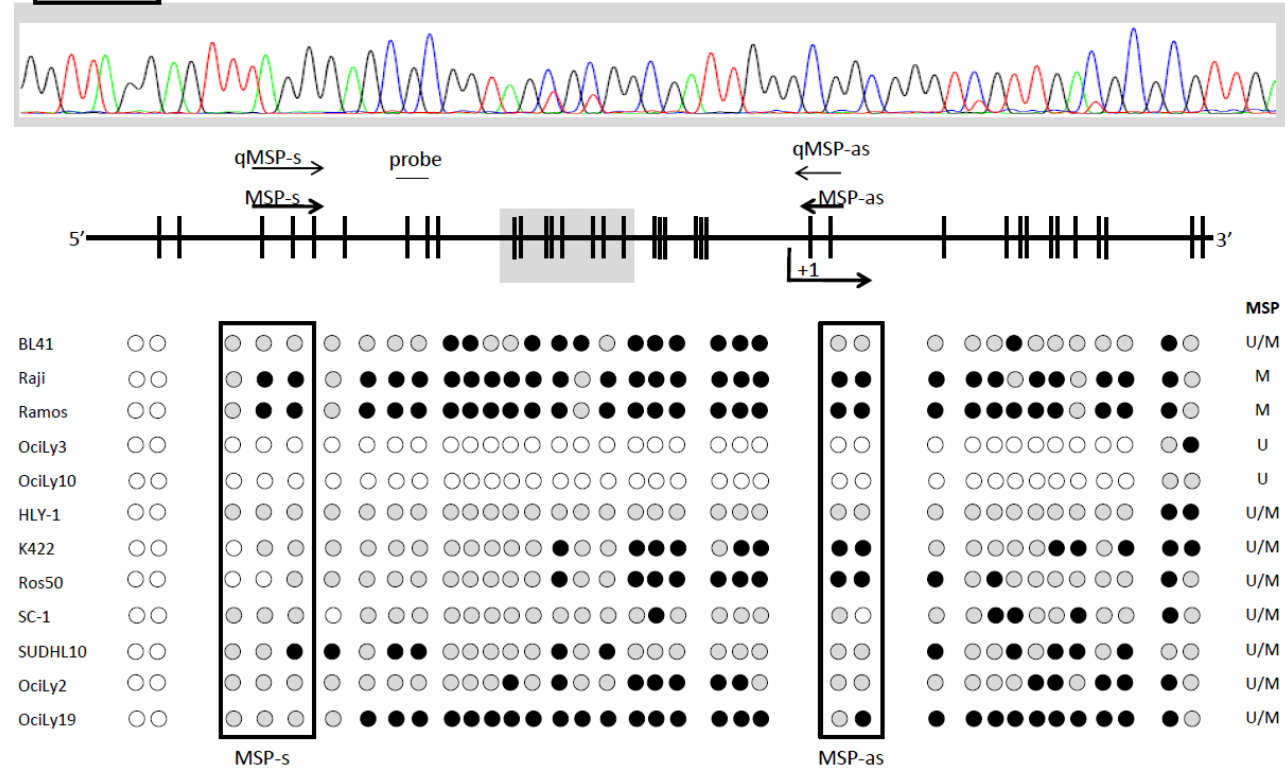

Supplement: Figure S2 — Bisulfite sequencing results of B-cell lymphoma cell lines. For each gene, the upper panel includes a representative part of the bisulfite sequencing electropherogram. Beneath is a schematic presentation of the individual CpG sites (vertical bars) in the area of transcription start amplified by the bisulfite sequencing primers. The qMSP and MSP primer and probe binding sites are indicated (arrows and straight line, respectively) along with the transcription start site (represented by +1). Twelve cell lines have been sequenced. Each sequenced CpG site is represented by a circle. The color white, gray and black indicates an unmethylated, partial methylated and fully methylated site, respectively. The column in the right side of each panel lists the methylation status for individual samples from MSP analyses. Abbreviations: U, unmethylated; M, methylated and U/M partially methylated. (PDF) [file pone.0079602.s002.pdf]
