# Supplementary material for: Identification of Highly Methylated Genes across Various Types of B-Cell Non-Hodgkin Lymphoma
Source: PLoS One. 2013 Nov 19;8(11):e79602. doi: 10.1371/journal.pone.0079602 (PMC3834187; doi:10.1371/journal.pone.0079602)
Supplement: Table S1 — PCR primer and probe sequences for methylation specific PCR, bisulfite sequencing, and quantitative methylation specific PCR. Abbreviations: BSP, bisulfite sequencing primers; M, methylated reaction; MSP, methylation specific PCR; qMSP, quantitative methylation specific PCR; U, unmethylated reaction. (PDF) [file pone.0079602.s003.pdf]

Table S1

| MSP Primer set | Sense primer | Antisense primer | Amplicon lenght |
|---|---|---|---|
| AREG M | TTTTGACGTTATGGGTTGC | CCTCGAACTATCCCGAAAC | 122 |
| AREG U | GGTTTTGATGTTATGGGTTGT | CCTCAAACTATCCCAAAACCT | 122 |
| C10orf118 M | TTACGTTGAAGATACGAACGC | ACTTCCCGACTCTCATACGA | 118 |
| C10orf118 U | GTTATGTTGAAGATATGAATGT | AACTTCCCAACTCTCATACAA | 118 |
| COMMD6 M | TTAGGTCGTTAGGGGTAGGAC | TTAACATCCAACGACGACTC | 123 |
| COMMD6 U | GTTAGGTTGTTAGGGGTAGGAT | CTTAACATCCAACAACAACTC | 123 |
| DSP M | AGGAGATTCGTTTCGTCGTC | CGAAATCGCTACTCGCTACC | 171 |
| DSP U | GGGAGGAGATTTGTTTTGTTGTT | TCACAAAATCACTACTCACTACC | 171 |
| DUSP1 M | TGA CGT TAT CGT TTC GTT AC | CGC GTT TAT ATA CGA CCT CT | 107 |
| DUSP1 U | GGG TGA TGT TAT TGT TTT GTT AT | AAA CAC ATT TAT ATA CAA CCT CT | 107 |
| EPHA4 M | CGGGTTTACGTTATCGGTC | CAACCATAATTCGCCGATAC | 129 |
| EPHA4 U | TGGTGGGTTTATGTTATTGGTT | CCCAACCATAATTCACCAATACC | 129 |
| FOS M | ATAAAAGTAGTGGTTGCGGC | ACACGATCACTACTCGTTCG | 110 |
| FOS U | GTTATAAAAGTAGTGGTTGTGGT | AACACAATCACTACTCATTCACT | 110 |
| FZD8 M | AGTGATTTCGTTGTTGGTC | ACAACGACACGATAATCTCTT | 104 |
| FZD8 U | GAAGTGATTTTGTTGTTGGTT | ACACAACAACACAATAATCTCTT | 104 |
| GNG7 M | GTCGGGACGTTAGGGTTC | GCGAACGCCCTTACCTAC | 142 |
| GNG7 U | TTTGTTGGGATGTTAGGGTTT | TTCACAAACACCCTTACCTAC | 142 |
| HERC1 M | ATTTCGTGAGATTCGTTTGAC | AAACTACGACGATCGCGT | 111 |
| HERC1 U | TAAATTTTGTGAGATTTGTTTGAT | AAACTACAACAATCACATCCT | 111 |
| JAM3 M | TTGTTATTATGGTGTCGGTTC | GCGACGCGACTTATAATC | 113 |
| JAM3 U | TTTTTGTTATTATGGTGTTGGTTT | ACAACACAACTTATAATCCCC | 113 |
| JUN M | TTTATTTCGTGAGTTTTCGC | ATTAACTCGCGTCGCTCT | 100 |
| JUN U | TATTTTATTTTGTGAGTTTTTGT | CCCATTAACTCACATCACTCT | 100 |

| MSP Primer set | Sense primer | Antisense primer | Amplicon lenght |
|---|---|---|---|
| JUNB M | TTTTGATAGTGACGCGAGTC | ACGAAAATACGCTCCGATTA | 100 |
| JUNB U | GTTTTTTGATAGTGATGTGAGTT | ACAAAAATACACTCCAATTAACC | 100 |
| JUND M | ATGTAAATGAGCGACGTTATTC | TCCACTAACGACGACTCCTA | 119 |
| JUND U | TTTATGTAAATGAGTGATGTTATTT | CTCCACTAACAACAACTCCTAC | 119 |
| KCNH2 M | TAGTATTGGATCGGTTGGGC | AACTCCTACAACGACGCTCC | 114 |
| KCNH2 U | TAGTATTGGATTGGTTGGGT | AACTCCTACAACAACACTCC | 114 |
| KLF2 M | GCGTTTATAAGGTTTGGGC | ACACCGATAAATAACTACCCGA | 100 |
| KLF2 U | GTGTTTATAAGGTTTGGGT | ACACCAATAAATAACTACCCAA | 100 |
| KLF9 M | TGAGTTAGGAGGTTCGGATC | TTCGCTACCTCGTACTACCC | 135 |
| KLF9 U | GTGAGTTAGGAGGTTTGGATT | TTTCACTACCTCATACTACCC | 135 |
| MTSS1 M | CGTTCGTTAGGTAGGGTGTC | CGCTAAACCGAAACCTCTC | 105 |
| MTSS1 U | GGTGTTTGTTAGGTAGGGTGTT | AACACTAAACCAAAACCTCTC | 105 |
| MYLIP M | GCGGTTAGGGAAATTTAGTC | CCTACGCTACGACAAAAACT | 167 |
| MYLIP U | GGTGGTTAGGGAAATTTAGTT | ACCTACACTACAACAAAAACT | 167 |
| NR4A2 M | AATCGGTTTTGTTCGTGAC | CTTACGCTACCGCTACCAA | 155 |
| NR4A2 U | TAAAATTGGTTTTGTTTGTGAT | TAACTTACACTACCACTACCAA | 155 |
| PITHD1 M | GTAGCGGCGTTTTTTACGAC | ACCCCCGCCGTAACTATAAC | 168 |
| PITHD1 U | GTAGTGGTGTTTTTTATGAT | ACCCCCACCATAACTATAAC | 168 |
| PTPN12 M | CGGGAGTTTAGTCGGTGTC | CCGACTACGATCGCTAACC | 175 |
| PTPN12 U | GTTGGGAGTTTAGTTGGTGTT | CCAACTACAATCACTAACCCC | 175 |
| RGS2 M | GAAGTCGGGGTCGTAGAC | AAAACGCGAATTAATCGACT | 123 |
| RGS2 U | TAAGAAGTTGGGGTTGTAGAT | AAAAACACAAATTAATCAACTAC | 123 |
| TMED2 M | ATTTTTGGTTTTTCGGAGTC | ATAAATTAACGAACTCCCGC | 112 |
| TMED2 U | AAAATTTTTGGTTTTTTGGAGTT | ATAAATTAACAAACTCCCACAAC | 112 |
| TRIM36 M | AGGTTGATTTGAGATGCGTC | CGACGCGAAAATCTAAAAAA | 172 |

| MSP Primer set | Sense primer | Antisense primer | Amplicon lenght |
|---|---|---|---|
| TRIM36 U | GGAAGGTTGATTTGAGATGTGTT | CTCAACACAAAAATCTAAAAAAA | 172 |
| TXNIP M | AGCGATTTTATTGATTGGTC | CAAAAAACGAAAACGTCTCT | 149 |
| TXNIP U | TATAGTGATTTTATTGATTGGTT | AAACAAAAAACAAAAACATCTCT | 149 |
| WBSCR16 M | AGGTTAGGTAAGAAATGTCGC | AAAACAAAAACGCTAACGAC | 160 |
| WBSCR16 U | GTAAGGTTAGGTAAGAAATGTTGT | AAAACAAAAACACTAACAACAAC | 160 |

| BSP Primer set | Sense primer | Antisense primer | Amplicon lenght |
|---|---|---|---|
| BSP DSP | GTGTGGTTTAGGYGTTTAGTG | AAACCTCCRTTACAACTCATATC | 347 |
| BSP FZD8 | GGAAGGGTTTTGTTTAGG | ATCTCTTAACATACCAACTCCTT | 322 |
| BSP KLF9 | ATTTAATTGTTTGGGAGTTGGA | CCCCTCCAATTAACCAATAAA | 369 |
| BSP MTSS1 | AGAATGGGYGTTTTTTTAGA | CCRCAAAAAAAAACCTACTAAT | 310 |
| BSP NR4A2 | AAGTTGGTTATTAAGGTGAAYG | CRACCRACTAAACAAACAA | 289 |

| qMSP Primer set | Sense primer | Antisense primer | Probe | Amplicon lenght |
|---|---|---|---|---|
| COMMD6 | GCGTTAGGTCGTTAGGGGTAGG | ACTTAACATCCAACGACGACTCG | GACGTTGTTTATGGAGGCG | 128 |
| DSP | GGAGGAGATTCGTTTCGTCG | AATCGCTACTCGCTACCTAAACCC | TTTCGACGTAGTTTTTTTGCGTT | 170 |
| FZD8 | GAAGTGATTTCGTTGTTGGTCG | GATACCCTTACACAACGACACGATAAT | GGCGTTGTTGTAGCGTT | 117 |
| KCNH2 | GTATTGGATCGGTTGGGCG | GCAACCCGCCCAAACTC | ATAGGTTCGGTTGTTGGC | 124 |
| KLF9 | CGTGAGTTAGGAGGTTCGGATC | CGTTTCGCTACCTCGTACTA | GTTACGGGATCGCGAGGT | 139 |
| MTSS1 | CGTTAGGTAGGGTGTCGATACGT | GCCTCCCTCTCCAAAGCCA | TTTCGCGTTGCGACGG | 99 |
| NR4A2 | TAAAATCGGTTTTGTTCGTGACG | ATTTATATAACTTACGCTACCGCTA | TAGGTCGGAAATATATTAAAGCGA | 168 |
