# Supplementary material for: Identification of Highly Methylated Genes across Various Types of B-Cell Non-Hodgkin Lymphoma
Source: PLoS One. 2013 Nov 19;8(11):e79602. doi: 10.1371/journal.pone.0079602 (PMC3834187; doi:10.1371/journal.pone.0079602)
Supplement: Table S2 — Threshold for calling samples as methylated or unmethylated based on the PMR value. For each gene, samples with PMR values equal to and greater than the indicated threshold were scored as methylated. Samples with lower values were scored as unmethylated. Abbreviations: PMR, percent methylated reference. (PDF) [file pone.0079602.s004.pdf]

Table S2

	Treshold
COMMD6	1
DSP	1
FZD8	1
KCNH2	4
KLF9	1
MTSS1	4
NR4A2	1
PPP1R14A	1
