# Supplementary material for: Identification of Highly Methylated Genes across Various Types of B-Cell Non-Hodgkin Lymphoma
Source: PLoS One. 2013 Nov 19;8(11):e79602. doi: 10.1371/journal.pone.0079602 (PMC3834187; doi:10.1371/journal.pone.0079602)
Supplement: Table S3 — Top 30 identified candidate genes for DNA methylation in NHL. Gene symbol, full gene name and chromosomal location are listed. All genes with CpG islands were subjected to MSP analysis in B-cell lymphoma cell lines. Genes in bold were further validated in clinical patient material by qMSP analysis. (PDF) [file pone.0079602.s005.pdf]

Table S3

**Gene**	**Gene name**	**Location**
AREG	Amphiregulin	4q13.3
C10orf118	Chromosome 10 open reading frame 118	10q25.3
CD69	CD69 molecule	12p13
COMMD6	COMM domain containing 6	13q22
**DSP**	Desmoplakin	6p24
DUSP1	Dual specificity phosphatase 1	5q34
EPHA4	EPH receptor A4	2q36.1
FOS	FBJ murine osteosarcoma viral oncogene homolog	14q24.3
**FZD8**	Frizzled family receptor 8	10p11.21
GNG7	Guanine nucleotide binding protein, gamma 7	19p13.3
HERC1	HECT and RLD domain containing E3 ubiquitin protein ligase family member 1	15q22
JAM3	Junctional adhesion molecule 3	11q25
JUN	Jun proto-oncogene	1p32-p31
JUNB	Jun B proto-oncogene	19p13.2
JUND	Jun D proto-oncogene	19p13.2
**KCNH2**	Potassium voltage-gated channel, subfamily H, member 2	7q36.1
KLF2	Kruppel-like factor 2	19p13.11
**KLF9**	Kruppel-like factor 9	9q13
**MTSS1**	Metastasis suppressor 1	8p22
MYLIP	Myosin regulatory light chain interacting protein	6p23-p22.3
**NR4A2**	Nuclear receptor subfamily 4, group A, member 2	2q22-q23
PITHD1	PITH domain containing 1	1p36.11
**PPP1R14A**	Protein phosphatase 1, regulatory subunit 14A	19q13.1
PTPN12	Protein tyrosine phosphatase, non-receptor type 12	7q11.23
RGS2	Regulator of G-protein signaling 2	1q31
SLC2A3	Solute carrier family 2 , member 3	12p13.3
TMED2	Transmembrane emp24 domain trafficking protein 2	12q24.31
TRIM36	Tripartite motif containing 36	5q22.3
TXNIP	Thioredoxin interacting protein	1q21.1
WBSCR16	Williams-Beuren syndrome chromosome region 16	7q11.23
